# A New Method of Remaining Useful Lifetime Estimation for a Degradation Process with Random Jumps

**DOI:** 10.3390/s25154534

**Published:** 2025-07-22

**Authors:** Yue Zhuo, Lei Feng, Jianxun Zhang, Xiaosheng Si, Zhengxin Zhang

**Affiliations:** Zhijian Laboratory, Rocket Force University of Engineering, Xi’an 710025, China; zhuoyue0519@outlook.com (Y.Z.); sixiaosheng@126.com (X.S.); zhangzx.2006@tsinghua.org.cn (Z.Z.)

**Keywords:** remaining useful life estimation, particle filtering, expectation maximization algorithm, state–space model, jump diffusion process

## Abstract

With the deepening of degradation, the stability and reliability of the degrading system usually becomes poor, which may lead to random jumps occurring in the degradation path. A non-homogeneous jump diffusion process model is introduced to more accurately capture this type of degradation. In this paper, the proposed degradation model is translated into a state–space model, and then the Monte Carlo simulation of the state dynamic model based on particle filtering is employed for predicting the degradation evolution and estimating the remaining useful life (RUL). In addition, a general model identification approach is presented based on maximization likelihood estimation (MLE), and an iterative model identification approach is provided based on the expectation maximization (EM) algorithm. Finally, the practical value and effectiveness of the proposed method are validated using real-world degradation data from temperature sensors on a blast furnace wall. The results demonstrate that our approach provides a more accurate and robust RUL estimation compared to CNN and LSTM methods, offering a significant contribution to enhancing predictive maintenance strategies and operational safety for systems with complex, non-monotonic degradation patterns.

## 1. Introduction

Lifetime or remaining useful life (RUL) prognostics, as an essential part of prognostics and health management (PHM), have attracted increasing attention and play an important role in many engineering systems [1,2,3]. In general, the common way of lifetime or RUL prognostics includes degradation process modeling and failure probability predicting [4,5,6]. Due to the difference in the degradation model, data-driven approaches can be classified into machine learning and statistics-based approaches [7]. Nowadays, statistical data-driven approaches that only rely on the available observed data and statistical models, one of the most popular degrading model methods, have been gaining in momentum and are applied in a variety of industrial assets [4,5]. In addition, based on the type of monitoring degradation data, the statistical data-driven approaches can be classified into the methods based on directly observed data and the methods based on indirectly observed data [4]. In this paper, we mainly focus on the statistical data-driven approaches for degrading systems.

In fact, owing to the complexity and uncertainty of the systems and their operating environment, the data directly observed from many industrial systems often cannot be obtained. A common way is to measure some given intermediate performance indicators, just like the temperature sensors for the thickness of the furnace wall [3,4,5,6,7,8,9,10,11]. As such, some outstanding random jumps exist in the degradation process. In addition, with the deepening of the systems’ deterioration, the stability of the systems becomes gradually worse, which makes the systems more vulnerable to random shock. It should be noted that if we ignore these random jumps, the degradation model is not suitable, which must affect the accuracy of the lifetime or RUL estimation [12]. In this paper, we mainly concentrate on degrading systems with random jumps.

To describe the character of these jumps, a direct way is to adopt the jump diffusion processes to describe such a degradation process. That is to say, the traditional diffusion process is used to describe the continuous degradation and the compound jump process to depict random jumps [13,14,15,16]. For example, the normal jump diffusion process is presented by Merton for a financial model in 1976 [17]. On this basis, Abundo provided an approximate FPT expression for the homogeneous jump diffusion process. Platen and Bruti-Liberati systematically discussed and reviewed the differential equation, numerical simulation method, and parameter identification [18]. However, as discussed in [19], if the jumps are described as a Gaussian variable, the analytical solution of the first passage time (FPT) cannot be obtained. Recently, Zhang et al. provided an approximate approach to derive the FPT, and the solution with the single integral form was attained [14]. Thus, it could be concluded that the lifetime or RUL estimation problem for the jump diffusion degradation process has not been well studied, and it is still challenging.

In this paper, we propose a non-homogeneous compound Poisson degradation process model to describe this kind of degradation trajectory. Unlike the traditional method, such as [20,21,22], we first translate the degradation model into the state–space model. Considering the nonlinear form of the degradation model, particle filtering (PF) is adopted to approximate the future degradation path and predict the lifetime and RUL. Compared with other classical Monte Carlo methods, it can reduce the size of the samples and the computational complexity. In addition, we propose a general identification method for such a degradation model and an iterative for linear degradation model based on an expectation maximization (EM) algorithm. To illustrate the applicability and rationality of our method, a practical case is provided.

The remaining sections are organized as follows. In Section 2, the motivating examples and problem formulation are given. In Section 3, a new stochastic model is proposed to describe the degradation path and the framework of the EM method for unknown model parameters is presented. Section 4 includes the main result of the RUL estimation. An illustrated example is presented to clarify the feasibility and applicability of the proposed model in Section 5. This paper is concluded in Section 6.

## 2. Motivating Examples and Formulation

### 2.1. Motivating Examples of Blast Furnace

**Figure 1:**
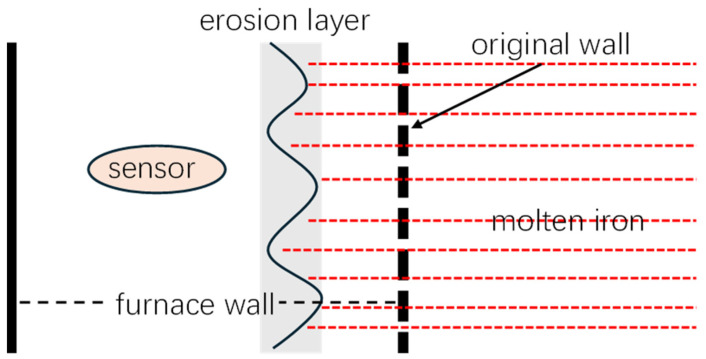
A chart of the furnace wall degradation.

Blast furnaces are large-scale degrading complex systems, and their degradation is caused by the erosion of molten iron. Due to the high cost of blast furnace downtime and the difficulty of directly measuring the furnace wall thickness, temperature sensors are commonly used in industrial practice to monitor the degree of wall degradation. In engineering practice, sensors are strategically placed at a suitable depth within the furnace wall to ensure safety [10,11]. A temperature reading that approaches that of molten iron (approx. 1400 °C) is a clear indicator that erosion has progressed to at least the sensor’s depth. Figure 1 illustrates the degradation process of the furnace wall. Consequently, once the temperature surpasses a predetermined threshold, the furnace must be taken offline for a major overhaul. Since this overhaul requires the complete reconstruction of the wall, it defines the end of the blast furnace’s lifespan.

**Figure 2:**
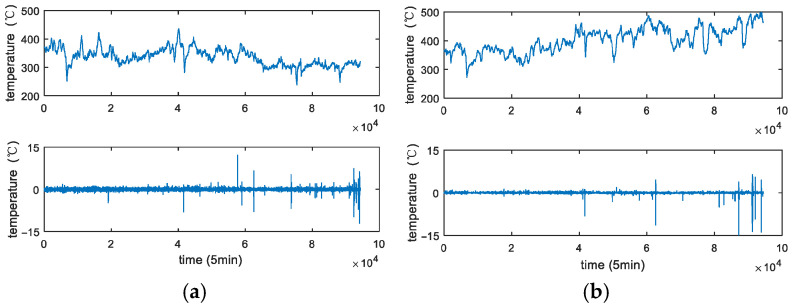
The actual degradation data from temperature sensors. (**a**) The actual degradation data from temperature sensor 1. (**b**) The actual degradation data from temperature sensor 2.

Figure 2 presents the degradation signals acquired from two sensors over a span of approximately one year, with a sampling rate of once every 5 min. It is noted that the blast furnace is just put into operation for a short time and the furnace wall is healthy. Because the erosion level of the furnace wall is low (the failure threshold is over 800 °C at least in engineering practice), no maintenance has been adopted for the furnace wall so far [12,13,14].

It is important to understand that the wall thickness degradation is a non-monotonic process. This occurs because two things happen at once: first, the molten iron erodes the wall, reducing its thickness. Second, it reacts to create unstable chemical compounds. These compounds are constantly being created and then breaking away from the wall. Because their ability to conduct heat is different from the wall’s, they cause the temperature sensor data to fluctuate randomly. This is precisely what we refer to as “random jumps” in our work [13,14].

Thus, from Figure 1, it can be found that the frequency of random jumps becomes higher over time. This is because as the furnace wall erodes and these complex compounds accumulate, the sensor becomes more sensitive to temperature variations. Neglecting these random jumps in favor of modeling only the continuous degradation component for RUL prognosis will inevitably introduce prediction bias. Such bias can lead to catastrophic failures and significant losses, thereby making it imperative to incorporate these stochastic events into the modeling framework.

### 2.2. Problem Formulation

Following the clarification in Section 1, we focus on the degradation model based on stochastic process. Define X(t) as the performance variable of degrading systems as time t, and as usual, X(t) is a stochastic process to depicting the degradation path. As per the definition in [23], the nonlinear degradation model could be described as follows:

(1) $$X(t)=X(0)+\int_{0}^{t}\mu (\tau ;\theta )d\tau +\sigma_{B}B(t)$$

where X(t) represses as a nonlinear Brownian motion with drift function μ(τ;θ) and diffusion coefficient $\sigma_{B}$ and B(t) denotes the standard Brownian motion. Moreover, its stochastic differential equation (SDE) can be presented as follows:

(2) $$dx(t)=\mu (\tau ;\theta )d\tau +\sigma_{B}dB(t)$$

In fact, the monitored data of degradation processes are not continuous, so let $X_{0:n}=\{x_{0},x_{1},\ldots ,x_{n}\}$ denote the degradation data at time $t_{i}$. Based on the Euler approximation [24], when Δt is small enough, the increment of Equation (2) has the following form:

(3) $$\Delta x_{i}=\mu (t_{i};\theta )\Delta t_{i}+\sigma_{B}B(\Delta t_{i})$$

where $\Delta x_{i}=x_{i+1}-x_{i}$ and $\Delta t_{i}=t_{i+1}-t_{i}$.

In order to model the degradation system with time-varying jumps, we defined two stochastic processes for description: one is used to describe the normal degradation process as Equations (1) and (2) shown, the other is used to depict the random jump process. Inspired by Merton’s model in [17], the jump diffusion degradation process model is defined as follows:

(4) $$X(t)=X(0)+\int_{0}^{t}\mu (\tau ;\theta )d\tau +\sigma_{B}B(t)+\sum_{i=1}^{N(t)}g_{i}$$

where $\left\{N(t),t\geq 0\right\}$ is the non-homogeneous Poisson process with locally integrable positive intensity function λ(t) and $g_{i}$ is an independent and identically Gaussian distributed random variable with mean $\mu_{g}$ and variance $\sigma_{g}^{2}$. Then the SDE of the jump diffusion process is given as follows:

(5) $$dX(t)=\mu (t;\theta_{\mu })dt+\sigma_{B}dB(t)+dY(t)$$

where $Y(t)=\sum_{j=0}^{N(t)}g_{i}$ is the non-homogeneous compound Poisson process. It is noted that if the intensity function λ(t) is constant, the probability of the jump occurrence will not be changed during the same time interval, i.e., the homogeneous compound Poisson process. If λ(t) is integrable, positive, and time-varying, the probability of the jump occurrence will increase over time. It should be noted that we assume the $g_{i}$ follows a Gaussian distribution. This choice is primarily motivated by the distribution’s mathematical simplicity and tractability, as well as its widespread use in mathematical modeling and machine learning. Furthermore, the effectiveness of the Gaussian assumption is validated in our subsequent case studies. Of course, since the increments of the random jumps can be both positive and negative, other distributions could also be used to model $g_{i}$. The derivation process in such cases would be analogous to the one presented in this paper.

In this paper, we focus on the lifetime under the concept of the FPT. That is to say, if the degradation process crosses the failure threshold, the systems fail at once. As defined in [16], the lifetime has the following form:

(6) $$T:=\inf\{t:X(t)\geq \xi \left|X(0)\leq \xi \right.\}$$

where T is the FPT, and is often defined as the lifetime and ξ denotes the failure threshold that is the constant value determined by engineering practical requirement. Let $f_{T}(t)$ and $F_{T}(t)$ express the probability density function (PDF) and cumulative distribution function (CDF) of T. Define $X_{0:k}=\{x_{0},x_{1},\ldots ,x_{k}\}$ as the degradation data at time $t_{0},t_{1},\ldots ,t_{k}$, then the RUL under concept of FPT is often defined as follows:

(7) $$L_{k}:=\inf\{l_{k}:X(t_{k}+l_{k})\geq \xi \left|X(t_{k})\leq \xi \right.\}$$

where $L_{k}$ denotes the RUL with PDF $f_{l_{k}}(t)$ and CDF $F_{l_{k}}(t)$.

Then, the remaining parts of this paper include two main goals: how to estimate the parameters in Equation (6) or Equation (7) through the conditional monitored data $X_{0:k}=\{x_{0},x_{1},\ldots ,x_{k}\}$ and how to derive the RUL via the degradation model and the estimated parameters.

## 3. Parameter Estimation for Degradation Model

In this section, we will introduce how to identify the proposed degradation model in a statistical way.

### 3.1. General Approach Based on MLE

Above all, for better illustration, the following Lemma 1 is given as follows:

**Lemma** **1** [25]**.** *Based on the definition of non-homogeneous Poisson process, we can define*

(8) $$\Lambda (t,t+\Delta t)=\int_{t}^{t+\Delta t}\lambda (\tau )d\tau$$

*Then the probability of jumps occurrence in *(t,t+Δt) *is shown as follows:*

(9) $$\mathrm{Pr}\left(N(t,t+\Delta t)=n\right)=\frac{{\left[\Lambda (t,t+\Delta t)\right]}^{n}}{n!}\exp\left(-\Lambda (t,t+\Delta t)\right)$$

Let $\theta_{\lambda }$ denote the parameter vector of Λ(t,t+Δt), then Λ(t,t+Δt) is rewritten as $\Lambda (t,t+\Delta t;\theta_{\lambda })$. In this way, it should be noticed that $\Theta =[\theta_{\lambda },\theta_{\mu },\sigma_{B},\mu_{g},\sigma_{g}]$ denotes all unknown parameters.

Then, as the definition in the above section, $X_{0:k}=\{x_{0},x_{1},\ldots ,x_{k}\}$ denotes all observed degradation data. Then we further define $\Delta X_{1:k}=\left\{\Delta x_{1},\Delta x_{2},\ldots ,\Delta x_{k}\right\}$ as the increment data, where $\Delta x_{i}=x_{i}-x_{i-1}$. According to the proposed degradation model, it is noted that the increment is independent and has Markov properties. Then we can formulate the likelihood as follows:

(10) $$\begin{array}{ll} L(\Theta \left|\Delta X_{1:k}\right.) & =\ln\prod_{i=1}^{k}p\left(\Delta x_{i};\Theta \right)\\ & =\sum_{i=1}^{k}\ln\sum_{n=0}^{+\infty }p\left(\Delta x_{i}\left|N(t_{i-1},t_{i})=n;\Theta \right.\right)\times \mathrm{Pr}\left(N(t_{i-1},t_{i})=n;\Theta \right)\\ & =\sum_{i=1}^{k}\ln\sum_{n=0}^{+\infty }\frac{\exp\left(-\Lambda (t_{i-1},t_{i})\right)\Lambda^{n}(t_{i-1},t_{i})}{\sqrt{2\pi (n\sigma_{g}^{2}+\Delta t_{i}\sigma_{B}^{2})}n!}\exp\left(-\frac{{\left(\Delta x_{i}-\int_{t_{i-1}}^{t_{i}}\mu (\tau ;\theta_{\mu })d\tau -n\mu_{g}\right)}^{2}}{2n\sigma_{g}^{2}+2\Delta t_{i}\sigma_{B}^{2}}\right) \end{array}$$

In this case, MLE is the direct method for estimating parameters, which has the following equation [26,27]:

(11) $$\begin{array}{ll} {\hat{\Theta }}_{k} & =\underset{\Theta }{\mathrm{argmax}}\;L(\Theta \left|X_{1:k}\right.)\\ & =\underset{\Theta }{\mathrm{argmax}}\;L(\Theta \left|\Delta X_{1:k}\right.) \end{array}$$

However, it is hard to derive the analytical solution of Equation (26) based on a traditional optimization algorithm. As such, the common way is choosing some heuristic optimization methods such as ant colony algorithm, the genetic algorithm, Tabu search algorithm, and so on. In addition, the existence and convergence of the optimization function is difficult to analyze.

### 3.2. Parameters Estimation Based on EM Algorithm

From Figure 2, it can be found that the degradation increments except the random jumps seem to be stationary, so the linear model is appropriate for practical data. In this subsection, we will introduce an iterative parameter estimation method based on the EM algorithm.

When the degradation model is linear, the likelihood can be translated into the following:

(12) $$\begin{array}{ll} L(\Theta \left|\Delta X_{1:k}\right.) & =\ln\prod_{i=0}^{k}p\left(\Delta x_{i};\Theta \right)\\ & =\sum_{i=0}^{k}\ln\sum_{n=0}^{+\infty }p\left(\Delta x_{i}\left|N(t_{i-1},t_{i})=n;\Theta \right.\right)\times \mathrm{Pr}\left(N(t_{i-1},t_{i})=n;\Theta \right)\\ & =\sum_{i=0}^{k}\ln\sum_{n=0}^{+\infty }\frac{\exp\left(-\Lambda (t_{i-1},t_{i})\right)\Lambda^{n}(t_{i-1},t_{i})}{\sqrt{2\pi (n\sigma_{Y}^{2}+\Delta t_{i}\sigma_{B}^{2})}n!}\exp\left(-\frac{{\left(\Delta x_{i}-\mu \Delta t_{i}-n\mu_{Y}\right)}^{2}}{2n\sigma_{Y}^{2}+2\Delta t_{i}\sigma_{B}^{2}}\right) \end{array}$$

Based on the character of the non-homogeneous Poisson process, the following results can be known:

(13) $$\begin{array}{ll} P\{N(t+\Delta t)-N(t)=1\} & =\lambda (t)\Delta t+o(\Delta t)\\ P\{N(t+\Delta t)-N(t)\geq 2\} & =o(\Delta t) \end{array}$$

where o(h) is asymptotic or little-o notation for o(h)/h→0 as h→0. That is to say, when the interval time Δt is small, the probability of P{N(t+Δt)−N(t)≥2} is very little. Thus, the following Assumption 1 is provided for simplifying the problem.

**Assumption** **1.** *If the sampling interval is small enough (*Δt*is small), it is assumed that the random jump only occurs once or not during sampling interval time *Δt* and these random jumps have the same expression. That is to say, we define *P{N(t+Δt)−N(t)=1}=λ(t)Δt *and *P{N(t+Δt)−N(t)=0}=1−λ(t)Δt*.*

In this way, we can find that the degradation increments have the following property,

(14) $$\Delta x_{i}=\alpha_{1,t_{i}}\left[\mu \Delta t_{i}+\sigma_{B}B(\Delta t_{i})\right]+\alpha_{2,t_{i}}\left[\mu \Delta t_{i}+\sigma_{B}B(\Delta t_{i})+\Delta g_{i}\right]$$

where $\alpha_{1,t_{i}}+\alpha_{2,t_{i}}=1$, $\alpha_{1,t_{i}},\alpha_{2,t_{i}}\in (0,1)$, $P\{\alpha_{1,t_{i}}=0,\alpha_{2,t_{i}}=1\}=\lambda (t)\Delta t$, $P\{\alpha_{1,t_{i}}=1,\alpha_{2,t_{i}}=0\}=1-\lambda (t)\Delta t$. Thus, $\Delta x_{i}$ follows a two-dimension Gaussian mixture distribution with mean $\mu \Delta t_{i}+\mu_{g}$ and variance $\Delta t_{i}\sigma^{2}+\sigma_{g}^{2}$. For better illustration, we define the following:

(15) $$\begin{array}{l} \mu_{0}=\mu \Delta t_{i},\mu_{1}=\mu \Delta t_{i}+\mu_{g},\cdot \cdot \cdot \mu_{j}=\mu \Delta t_{i}+j\mu_{g}\\ \sigma_{0}^{2}=\Delta t_{i}\sigma_{B}^{2},\sigma_{1}^{2}=\Delta t_{i}\sigma_{B}^{2}+\sigma_{g}^{2},\cdot \cdot \cdot \sigma_{j}^{2}=\Delta t_{i}\sigma_{B}^{2}+j\sigma_{g}^{2} \end{array}$$

Clearly, the probability of a jump occurring, as governed by $\frac{1}{i}[\Lambda (t,t+\Delta t)]$, decreases as i increases. For i≥2, we consider this probability to be negligible. Furthermore, our statistical analysis of the blast furnace degradation case shows that even when the sampling interval is doubled to 10 min, the probability of two or more jumps occurring within an interval is merely 0.000159, which further validates the effectiveness of Assumption 1.

It is noted that if the interval time is fixed, i.e., $\Delta t_{i}=\Delta t$, $\left\{\Delta x_{1},\Delta x_{2},\ldots ,\Delta x_{k}\right\}$ can be regarded as the observation samples of a two-dimension Gaussian mixture distribution with mean $\mu_{1}$ and $\mu_{2}$ and variance $\sigma_{1}^{2}$ and $\sigma_{2}^{2}$. In addition, the two weights (i.e., $w_{1,k}$ and $w_{2,k}$) of such a two-dimension Gaussian mixture distribution at time $t_{k}$ can be written as follows [27]:

(16) $$\begin{array}{l} w_{1,k}=1-\sum_{i=1}^{k}\lambda (t_{i})\Delta t_{i}\\ w_{2,k}=\sum_{i=1}^{k}\lambda (t_{i})\Delta t_{i} \end{array}$$

In this case, it is natural for the algorithm to estimate the parameters of such a two-dimensional mixture of Gaussian distribution.

Firstly, let $Y_{1:k}=\{y_{1},y_{2},\ldots ,y_{k}\}$, $y_{i}\in \{0,1\}$ denote the latent variables which determine the degradation data from which the model originates, e.g., if $y_{i}=1$, it means $\Delta x_{i}\sim N(\mu_{1},\sigma_{1}^{2})$ and if $y_{i}=0$, it means $\Delta x_{i}\sim N(\mu_{2},\sigma_{2}^{2})$. Then the complete-data likelihood function holds the following form:

(17) $$\ln L(\Xi \left|\Delta X,Y\right.)=\ln\prod_{i=1}^{k}p(\Delta x_{i},y_{i}\left|\Xi \right.)=\sum_{i=1}^{k}\ln\left(w_{y,k}p_{y,i}(x_{i}\left|\theta_{y}\right.)\right)$$

where ${\hat{\Xi }}_{k}^{\left(i\right)}=[{\hat{w}}_{k}^{\left(i\right)},{\hat{\mu }}_{1,k}^{\left(i\right)},{\hat{\sigma }}_{1,k}^{\left(i\right)},{\hat{\mu }}_{2,k}^{\left(i\right)},{\hat{\sigma }}_{2,k}^{\left(i\right)}]$, $\theta_{y}=[\mu_{y},\sigma_{y}]$, $y_{i}\in \{0,1\}$. Generally, the EM method mainly includes E-step and M-step [28].

E-step:

(18) $$\begin{array}{ll} \ln L(\Xi_{k}\left|{\hat{\Xi }}_{k}^{\left(i\right)}\right.) & =E_{Y\left|\Delta X_{1:k},{\hat{\Xi }}_{k}^{\left(i\right)}\right.}\left\{\ln p(\Delta X_{1:k},Y_{1:k}\left|{\hat{\Xi }}_{k}^{\left(i\right)}\right.)\right\}\\ & =\sum_{y}\ln L({\hat{\Xi }}_{k}^{\left(i\right)}\left|\Delta X_{1:k},y\right.)p(y\left|\Delta X_{1:k},{\hat{\Xi }}_{k}^{\left(i\right)}\right.)\\ & =\sum_{y}\sum_{i=1}^{k}\ln\left(w_{y,i}p_{y}(\Delta x_{i}\left|\theta_{y}\right.)\right)p(y\left|\Delta x_{i},{\hat{\Xi }}_{k}^{\left(i\right)}\right.)\\ & =\sum_{y=1}^{2}\sum_{i=1}^{k}\ln\left(w_{y,i}p_{y}(\Delta x_{i}\left|\theta_{y}\right.)\right)p(y\left|\Delta x_{i},{\hat{\Xi }}_{k}^{\left(i\right)}\right.) \end{array}$$

where

(19) $$\begin{array}{l} p(y\left|\Delta x_{j},{\hat{\Xi }}^{\left(i\right)}\right.)=\frac{p(y,\Delta x_{j}\left|{\hat{\Xi }}^{\left(i\right)}\right.)}{\sum_{y^{\prime }=1}^{2}p(y^{\prime },\Delta x_{j}\left|{\hat{\Xi }}^{\left(i\right)}\right.)}=\frac{{\hat{w}}_{y,k}^{\left(i\right)}p_{y}(\Delta x_{j}\left|{\hat{\Xi }}^{\left(i\right)}\right.)}{\sum_{m^{\prime }=1}^{2}{\hat{w}}_{y^{\prime },k}^{\left(i\right)}p_{y^{\prime }}(\Delta x_{j}\left|{\hat{\Xi }}^{\left(i\right)}\right.)}\\ p_{y}(\Delta x_{j}\left|{\hat{\Xi }}^{\left(i\right)}\right.)=\frac{1}{\sqrt{2\pi \Delta t{\hat{\sigma }}_{y,k}^{2(i)}}}\exp\left(-\frac{{\left(\Delta x_{j}-{\hat{\mu }}_{y,k}^{\left(i\right)}\right)}^{2}}{2\Delta t{\hat{\sigma }}_{y,k}^{2(i)}}\right) \end{array}$$

M-step:

Then let $\frac{\partial \ln L(\Xi_{k}\left|{\hat{\Xi }}_{k}^{\left(i\right)}\right.)}{\partial \Xi_{k}}=0,$ the closed-form solutions of ${\hat{\Xi }}_{k}^{\left(i+1\right)}$ can be obtained.

(20) $$\begin{array}{l} {\hat{\mu }}_{y,k}^{\left(i\right)}=\frac{\sum_{j=1}^{k}\Delta x_{j}p(y\left|\Delta x_{j},{\hat{\Xi }}^{\left(i-1\right)}\right.)}{\Delta t\sum_{j=1}^{k}p(y\left|\Delta x_{j},{\hat{\Xi }}^{\left(i-1\right)}\right.)},\\ {\hat{\sigma }}_{y,k}^{2(i)}=\frac{\sum_{j=1}^{k}\left(\Delta x_{j}-{\hat{\mu }}_{m,k}^{\left(i\right)})p(y\left|\Delta x_{j},{\hat{\Xi }}^{\left(i-1\right)}\right.\right)}{\Delta t\sum_{j=1}^{k}p(y\left|\Delta x_{j},{\hat{\Xi }}^{\left(i-1\right)}\right.)},\left(y=1\;or\;2\right)\\ {\hat{w}}_{y,k}^{\left(i\right)}=\frac{1}{k}\sum_{j=1}^{k}p(y\left|\Delta x_{j},{\hat{\Xi }}^{\left(i-1\right)}\right.), \end{array}$$

where

(21) $$\begin{array}{l} p(y\left|\Delta x_{j},{\hat{\Xi }}^{\left(i-1\right)}\right.)=\frac{{\hat{c}}_{y,k}^{\left(i-1\right)}p_{y}(\Delta x_{j}\left|{\hat{\Xi }}^{\left(i-1\right)}\right.)}{\sum_{y^{\prime }=1}^{2}{\hat{c}}_{y^{\prime },k}^{\left(i-1\right)}p_{y^{\prime }}(\Delta x_{j}\left|{\hat{\Xi }}^{\left(i-1\right)}\right.)}\\ p_{y}(\Delta x_{j}\left|{\hat{\Xi }}^{\left(i-1\right)}\right.)=\frac{1}{\sqrt{2\pi \Delta t{\hat{\sigma }}_{y,k}^{2(i-1)}}}\exp\left(-\frac{{\left(\Delta x_{j}-{\hat{\mu }}_{y,k}^{\left(i-1\right)}\right)}^{2}}{2\Delta t{\hat{\sigma }}_{y,k}^{2(i-1)}}\right) \end{array}$$

In this way, we can obtain the estimates of ${\hat{\Xi }}_{k}=[{\hat{w}}_{k},{\hat{\mu }}_{1,k},{\hat{\sigma }}_{1,k},{\hat{\mu }}_{2,k},{\hat{\sigma }}_{2,k}]$ and then the estimates of $\mu ,\sigma_{B},\mu_{g},\sigma_{g}$ can be derived based on the relationship in Equation (15). As discussed before, let $\theta_{\lambda }$ denote the known parameters of λ(t), then we can derive the ${\hat{\theta }}_{A}$ incorporating the estimates of $\left\{{\hat{w}}_{y,1},{\hat{w}}_{y,2},\ldots ,{\hat{w}}_{y,k}\right\}$, which can be concluded as solving the over-determined equations.

(22) $$\left\{\begin{array}{l} \sum_{i=1}^{1}\lambda (t_{i})\Delta t={\hat{w}}_{y,1}\\ \sum_{i=1}^{2}\lambda (t_{i})\Delta t={\hat{w}}_{y,2}\\ \begin{array}{ccc} & & \vdots \end{array}\\ \sum_{i=1}^{k}\lambda (t_{i})\Delta t={\hat{w}}_{y,k} \end{array}\right.$$

where the over-determined equations can be solved via least squares method and heuristic optimization methods [29].

**Remark** **1.** *Unlike utilizing the traditional EM method directly, the heuristic optimization methods are only used once at each sampling time, which can reduce the running time and improve the online capacity.*

## 4. RUL Estimation Based on State-Space Model

**Figure 3:**
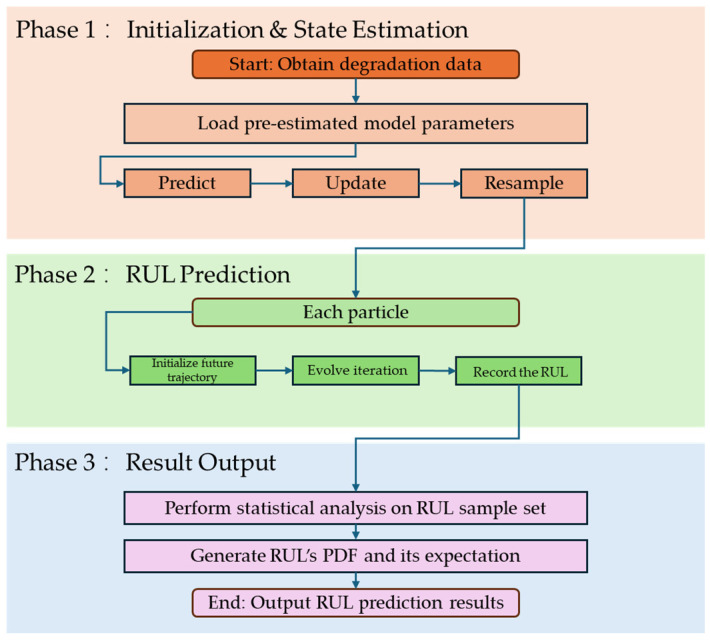
A flowchart of the particle filter estimation method.

In this section, we further introduce how to obtain the RUL of the proposed degradation model. As discussed in many research studies, it is hard to derive the analytical solution of the lifetime subject to the proposed jump diffusion degradation process model. Therefore, we provide a Monte Carlo approach to derive the RUL under the concept of the FPT. Figure 3 illustrates the flowchart of the particle filter estimation method proposed in this Section.

According to the proposed degradation model and its stochastic differential equation, we can utilize the Euler approximation method to translate the continuous degradation process into a state–space model, as shown in the following equation.

(23) $$\begin{array}{l} x_{1,k}=x_{1,k-1}+\int_{t_{k-1}}^{t_{k}}u(\tau ;\theta )d\tau +\sigma_{B}B(\Delta t_{k})\\ N(t_{k})=N(t_{k-1})+Po(\Delta t_{k};\theta_{\lambda })\\ x_{k}=x_{1,k}+N(t_{k})\times g_{k} \end{array}$$

where $Po(\Delta t_{k};\theta_{\lambda })$ is a random variable and follows a Poisson distribution with interval time $\Delta t_{k}$ and parameters $\theta_{\lambda }$. It is worth mentioning that $x_{1,k}$ and $N(t_{k})$ can be regarded as the system state, which reflects the continuous degradation and the number of random jumps accordingly. In addition, $x_{k}$ is the observed degradation data, which can be treated as the measurement output. In this case, we can build a nonlinear and non-Gaussian state-space model as follows:

(24) $$\begin{array}{ll} z_{k} & =f_{k}(z_{k-1},\nu_{k-1})\\ x_{k} & =h_{k}(z_{k},g_{k}) \end{array}$$

where $z_{k}=\left[\begin{array}{l} x_{1,k}\\ N(t_{k}) \end{array}\right]$ denotes the system state; $\nu_{k}=\left[\begin{array}{l} \sigma_{B}B(\Delta t_{k})\\ Po(\Delta t_{k};\theta_{\lambda }) \end{array}\right]$ represents the independent and identically distributed process noise sequence; and $g_{k}$ denotes the measurement noise sequence.

It is noted that if we want to simulate the RUL at time $t_{k}$ based on the Monte Carlo approach, it is essential to estimate the system state, i.e., $x_{1,k}$ and $N(t_{k})$. However, since the state–space model is nonlinear and non-Gaussian, the traditional Kalman filtering is not available for this state–space model. As such, it is natural to adopt particle filtering to estimate the system state and predict the future system state and measurement output owing to its good feature for nonlinear and non-Gaussian system.

It is well-known that the key of the particle filtering is to approach the posterior PDF through a given set of particles ${\left\{z_{k}^{\left(j\right)}\right\}}_{j=1}^{n}$, where n denotes the number of the particles. Generally, the standard particle filtering includes the following steps:

(1) Let k=0 and then generate the initial particles $z_{0}^{\left(j\right)}\sim p(z_{0})$, i=1,2,…,n

(2) Define and assign the weight of each particle,

(25) $$\omega_{k}^{\left(j\right)}=\omega_{k-1}^{\left(j\right)}p(x_{k}|z_{k-1}^{\left(j\right)})=\omega_{k-1}^{\left(j\right)}\frac{p(x_{k}|z_{k-1}^{\left(j\right)})p(z_{k}^{\left(j\right)}|z_{k-1}^{\left(j\right)})}{q(z_{k}^{\left(j\right)}|z_{0:k-1}^{\left(j\right)},x_{1:k})}$$

and then normalize the weights we can obtain,

(26) $$\omega_{k}^{\left(j\right)}=\frac{\omega_{k}^{\left(j\right)}}{\sum_{j=1}^{n}\omega_{k}^{\left(j\right)}}$$

(3) If $n_{eff}=\frac{n}{\sum_{j=1}^{n}{\left[\omega_{k}^{\left(j\right)}\right]}^{2}}$ is less than n, do the re-sampling precedence based on the $\omega_{k}^{\left(j\right)}$ and obtain the new sample ${\tilde{z}}_{k}^{\left(j\right)}\sim p(z_{0:k}^{\left(j\right)}|x_{1:k})$ with new weights ${\tilde{\omega }}_{k}^{\left(j\right)}=\frac{1}{n}$ to replace the previous one; else ${\tilde{z}}_{k}^{\left(j\right)}\sim {\tilde{z}}_{k}^{\left(j\right)}$, and ${\tilde{\omega }}_{k}^{\left(j\right)}=\omega_{k}^{\left(j\right)}$.

(4) Then the estimate of the state of the system can be calculated as follows:

(27) $${\hat{z}}_{k}=\sum_{j=1}^{n}{\tilde{\omega }}_{k}^{\left(j\right)}{\tilde{z}}_{k}^{\left(j\right)}$$

In this way, we can obtain the estimate of the system state in this degradation state–space model. Then, based on the state–space model, we can predict the RUL based on the following equation:

(28) $$L_{k}=\sum_{j=1}^{n}{\tilde{\omega }}_{k}^{\left(j\right)}L_{k}^{\left(j\right)}$$

where $L_{k}^{\left(j\right)}$ denotes the FPT of the *j*-th particle reaching the failure threshold. It is worth mentioning that in the FPT of each particle exists some predicted bias due to the Euler approximation method. 

## 5. Case Study

In this section, two examples are provided for illustration: (1) the numerical simulation is adopted to verify the accuracy of the parameter estimation and estimate of the state–space model; (2) the actual degradation data of the furnace wall is used to illustrate the feasibility of the proposed model.

### 5.1. Numerical Example

In this subsection, we mainly introduce a numerical simulation to illustrate the proposed model identification method based on the EM algorithm and state–space estimation approach based on particle filtering. As discussed before, the frequency of the jump occurrence becomes larger, so the form of λ(t) in compound Poisson process should not be a fixed value. In addition, what should be noticed is that the frequency of the jump occurrence in practical cases will not increase with the deepening of the degradation all the time. As such, in this paper, we define the expression of λ(t) as shown in the following equation:

(29) $$\lambda (t)=\theta_{\lambda 3}\Phi \left(\theta_{\lambda 2}+\theta_{\lambda 1}t\right)$$

where $\Phi \left(\cdot \right)$ denotes the CDF of the norm Gaussian distribution. In addition, we assume the continuous degradation process is linear, the degradation process model can be written as follows:

(30) $$X(t)=X(0)+\mu t+\sigma_{B}B(t)+\sum_{i=1}^{N(t)}g_{i}$$

The initial parameters are shown in Table 1.

**Figure 4:**
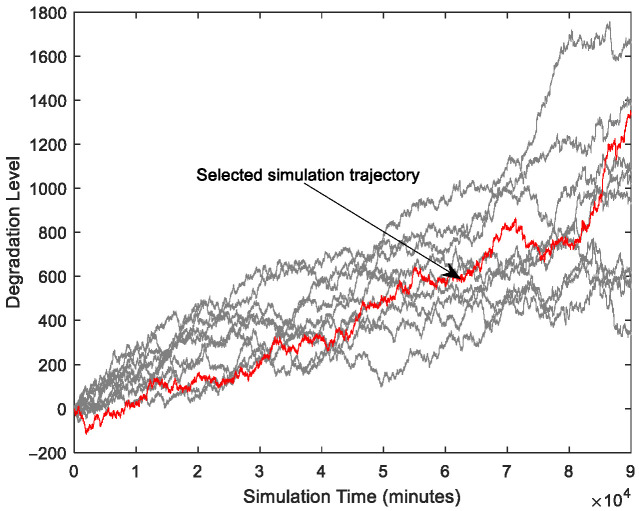
Degradation data from simulation.

**Figure 5:**
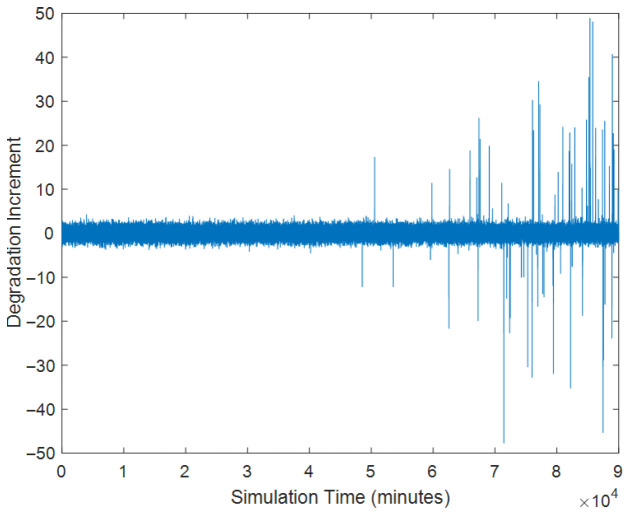
The degradation increments.

Then based on the given parameters, we define the interval time as 1 and then can obtain the degradation path and the increment process are shown in Figure 4 and Figure 5.

As shown in Figure 4, the simulated degradation trajectory does not monotonically increase or decrease over time. This is due to the inherent stochastic effects of the model. From Figure 5, it is noted that the frequency of the random jump occurrence is higher over time. Then, based on the proposed parameter estimation approach, we can obtain the parameter estimates.

From Table 2, it can be found that the estimation error decreases gradually. However, unlike $\mu_{1}$, $\mu_{2}$, $\sigma_{1}$, and $\sigma_{2}$, $\theta_{\lambda 1}$, $\theta_{\lambda 2}$, and $\theta_{\lambda 3}$ are estimated via solving the over-determined equation.

**Figure 6:**
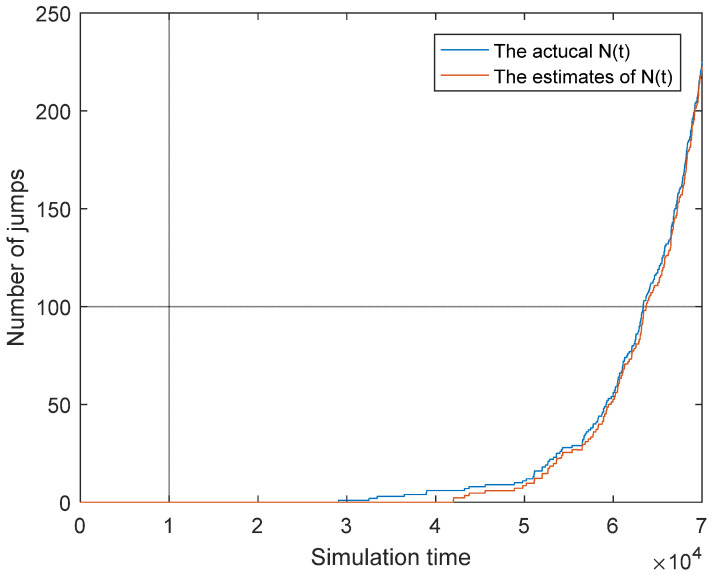
The estimates, actual value, and theoretical value of N(t).

Moreover, we further estimate the number of the jumps, i.e., *N*(*t*) in Equation (23) based on particle filtering. Figure 6 shows the comparison between the actual value and its estimates.

From Figure 6, we can see that the error between the estimates and the actual value is small, but the error between the actual value and the theoretical value always existed, which is inevitable in the numerical simulations.

### 5.2. Practical Example for Furnace Wall

As introduced in Section 2, it is noted that the furnace wall is a typical degradation process. In this subsection, we choose the data whose degradation feature is obvious, as shown in Figure 2. In practice, the failure threshold is usually set as 800 °C. Unfortunately, the degradation data of this furnace wall is below the soft failure threshold. For better illustration, we choose the upper limit of alert, i.e., 500 °C as the soft failure threshold in this paper.

From Figure 2, it can be found that the degradation increments except the random jumps seem to be stationary, so the linear model is appropriate for the practical data. That is to say, we choose the degradation model as Equation (27) to describe the degradation process of furnace wall.

Based on the proposed model identification method, we can obtain the estimates of all parameters as shown in the following table.

**Figure 7:**
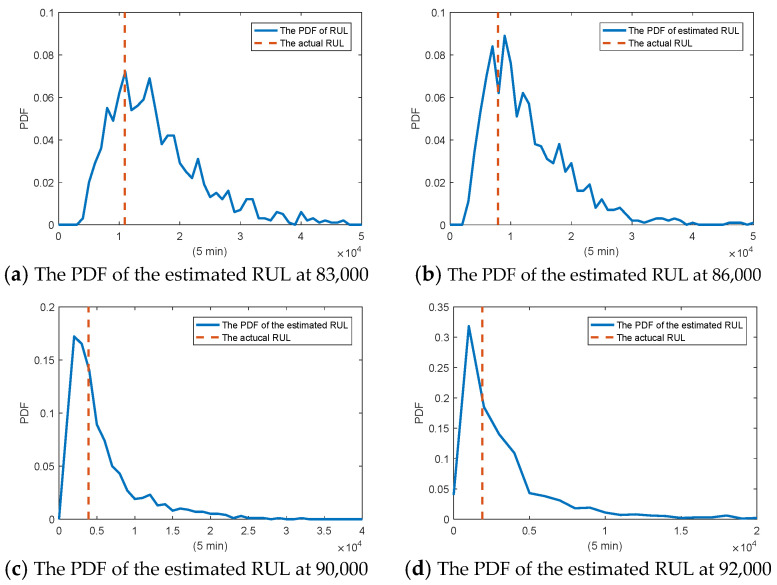
The PDF of the estimated RUL at different testing times.

From Table 3, it is noteworthy that $\sigma_{g}$ is much larger than $\sigma_{B}$, which can illustrate the existence of the random jumps. Then, combing the estimates of the parameters, we can predict the RUL based on the particle filtering method. Figure 7 shows the estimate of RUL at different testing times. Empirical tests on an Intel Core i9-13900H processor produced by Intel Corporation, Santa Clara, CA, USA. show that with 200 particles, the prediction time is 168 s, which is well below the 5-min sampling interval. This demonstrates the method’s feasibility for real-time industrial applications. It is important to note, however, that this prediction is performed on a large-scale dataset within a two-dimensional state–space. Therefore, while the prediction time is acceptable, it is still relatively slow. Future work could focus on reducing the number of particles to further decrease the computation time.

**Figure 8:**
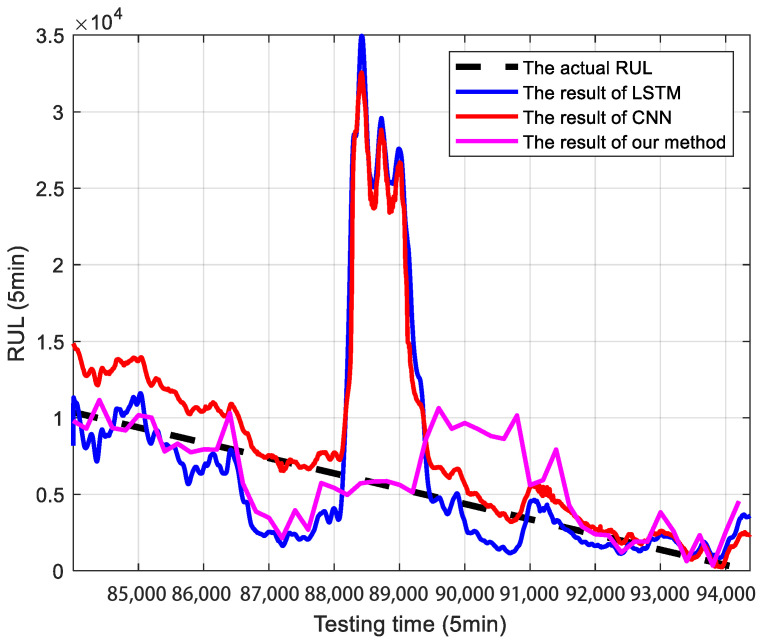
Comparison of RUL prediction results between the proposed method, CNN, and LSTM.

Figure 8 compares the results of our proposed method with those of the CNN and LSTM methods. Table 4 presents the quantitative metrics of our proposed method for RUL prediction, in comparison with the CNN and LSTM methods.

From the above figures and table, it is interesting to see that our results could predict the RUL better than the traditional methods. As shown in Table 4, our proposed method significantly outperforms both the CNN and LSTM baselines in RUL prediction. After correcting for an apparent swap in our method’s metric values, our model achieves an MAE of 1.8541 × 10^3^ and an RMSE of 2.7826 × 10^3^. These results represent a performance improvement of over 42% in MAE and 56% in RMSE compared to the next-best model (CNN). This demonstrates the enhanced accuracy and robustness of our approach. Thus, it can be summarized that it is essential to consider such random jumps in degradation modeling and RUL prediction.

## 6. Discussion

This paper proposes a non-homogeneous jump diffusion model to describe complex degradation processes involving time-varying random jumps. By reformulating it into a state–space form and integrating particle filtering (PF) and the expectation maximization (EM) algorithm, the model demonstrates superior accuracy and robustness in RUL prediction compared to traditional methods.

However, the proposed method has its limitations. Its key assumptions—a single jump per interval and a Gaussian jump magnitude—may not be suitable for all applications. Furthermore, the high computational cost associated with the particle filter and EM algorithm restricts the model’s use in high-dimensional or demanding real-time scenarios.

Future research directions include: (1) relaxing the model assumptions, such as considering multiple jumps and non-Gaussian distributions; (2) employing more efficient filtering algorithms to reduce the computational burden; and (3) extending the framework to multivariate degradation systems and other engineering domains.

## Figures and Tables

**Table 1:** The setting value of parameters for simulation.

Parameter	μ	$\mu_{g}$	$\sigma_{B}$	$\sigma_{g}$	$\theta_{\lambda 1}$	$\theta_{\lambda 2}$	$\theta_{\lambda 3}$
value	0.01	20	1	1	5/100,000	−5	0.02

**Table 2:** Parameter estimates at different time.

Sampling Interval	$\mu_{1}$	$\mu_{g}$	$\sigma_{B}$	$\sigma_{g}$	$\theta_{\lambda 1}$	$\theta_{\lambda 2}$	$\theta_{\lambda 3}$
t = 55,000	0.016	26.5	1.262	22.4	0.000079	−6.05	0.023
t = 60,000	0.014	22.7	0.988	19.6	0.000065	−5.23	0.021
t = 65,000	0.011	21.4	1.134	20.5	0.000069	−5.74	0.024
t = 70,000	0.011	19.3	0.996	20.1	0.000061	−5.68	0.022

**Table 3:** The estimates of all parameters in practical case.

μ	$\mu_{g}$	$\sigma_{B}$	$\sigma_{g}$	$\theta_{1}$	$\theta_{2}$	$\theta_{3}$
$9.023\times 10^{-4}$	0.0212	0.238	1.23	$5.631\times 10^{-6}$	−1.4634	0.0222

**Table 4:** Comparison of RUL prediction metrics for the proposed method vs. CNN and LSTM.

	Our Method	LSTM	CNN
MAE (10^3^)	2.7826	3.5505	3.2198
RMSE (10^3^)	1.8541	6.9281	6.3769

## Data Availability

The data that support the findings of this study are available from the corresponding author upon reasonable request.

## Abbreviations

The following abbreviations are used in this manuscript:

RUL	remaining useful life
MLE	maximization likelihood estimation
EM	expectation maximization
PF	particle filtering
FPT	first passage time
PDF	probability density function
CDF	cumulative density function
